# Inclusion of oral health teams in primary health care promotes early diagnosis of oral and oropharyngeal cancers: a nationwide study

**DOI:** 10.1186/s12903-021-01664-3

**Published:** 2021-06-18

**Authors:** Deborah Gomes de Miranda Vargas, Livia Fernandes Probst, Amanda Ramos da Cunha, Elaine Pereira da Silva Tagliaferro, Edílson José Zafalon, Paulo Zárate-Pereira, Alessandro Diogo De-Carli

**Affiliations:** 1 Postgraduate Program in Family Health (PPGSF), Federal University of Mato Grosso Do Sul (UFMS), Campo Grande, Mato Grosso Do Sul Brazil; 2 Faculty of Dentistry (FAODO), Federal University of Mato Grosso Do Sul (UFMS), Campo Grande, Mato Grosso Do Sul Brazil; 3 Piracicaba Dental School (FOP), State University of Campinas (UNICAMP), Piracicaba, São Paulo, Brazil; 4 School of Dentistry, Faculty of Dentistry, Federal University of Rio Grande Do Sul (UFRGS), Porto Alegre, Rio Grande do Sul, Brazil; 5 School of Dentistry (FOAr), São Paulo State University (UNESP), Araraquara, São Paulo, Brazil

**Keywords:** Oral neoplasms, Oropharyngeal neoplasms, Primary Health Care, Early cancer detection, Study of time series

## Abstract

**Background:**

Oral and oropharyngeal cancers are considered important public health problems worldwide. This study aims to analyze the association between late diagnosis of oral and oropharyngeal cancers in Brazil and the contextual indicators of socioeconomic variables and coverage of Primary Health Care (PHC), and to assess the temporal trend of late diagnosis.

**Methods:**

In this cross-sectional observational study, secondary data were evaluated with a time series analysis. All Brazilian cities that reported at least one case of oral and oropharyngeal cancers each year in the period between 2000 and 2013 were included; and the staging was analyzed by calculating the ratio risk for late diagnosis for each municipality. The association between staging and socioeconomic variables and offer of PHC was calculated using multiple linear regression. The time trend of the risk ratio for late-stage diagnosis was calculated using the Prais–Winsten method.

**Results:**

One hundred and sixty Brazilian municipalities had at least one annual case of oral and oropharyngeal cancers notified to the INCA hospital system between 2000 and 2013. The adjusted model showed that the higher the Gini value (greater social inequality) and the lower the HDI value (less human development) was, the higher was the number of tumors diagnosed at a late stage, considering the size of the tumor. A greater risk for late diagnosis was identified, as early as at the stage of lymph node involvement, when there was a higher level of social inequality and lower level of coverage by Oral Health Teams (OHT) in PHC. The greater the social inequality, the greater was the risk of late diagnosis, as early as in the stage of metastasis.

**Conclusions:**

We concluded that, during the evaluated period, there was an increase in the number of cases diagnosed at the most advanced stage. Furthermore, there was association between higher levels of social inequality and an increase in the proportion of late diagnosis of oral and oropharyngeal cancers. In addition, the inclusion of Oral Health Teams in Primary Health Care promoted the early diagnosis of these types of cancers.

**Supplementary Information:**

The online version contains supplementary material available at 10.1186/s12903-021-01664-3.

## Background

Oral and oropharyngeal cancers are considered important public health problems worldwide, due to their high incidence, mortality and the high clinical-care costs associated with them [1–3]. Prevention of the main risk factors combined with early detection are the most effective means of improving survival and reducing the morbidity and mortality rates associated with diseases. Therefore, the time between onset of symptoms, diagnosis and implementation of adequate treatment directly affects the progression of disease and prognosis [4].

The timely diagnosis and adequate treatment, among other factors, are associated with the organization and quality of health services offered. In this sense, Brazil has experienced an increase in the population’s access to oral health care [5, 6] after the Unified Health System (SUS, *Sistema Único de Saúde*) was created. The National Oral Health Policy (PNSB, *Política Nacional de Saúde Bucal*), created in 2004, consolidated Primary Health Care (PHC), and incorporated dental care into it, at three levels of care [7]. At the PHC level, Oral Health Teams (OHTs) include a Dental Surgeon and an Oral Health Assistant only; or in addition to these two professionals, an Oral Health Technician. Based on the concept of multiprofessional work, the OHT must recognize local vulnerabilities and the possibilities of intersectoral partnerships to achieve comprehensive care and work based on the principles and values of health promotion [7, 8].

Implementation of Oral Health Teams in the Family Health Strategy (FHS) have allowed the expansion of access to, and qualification of oral health care in SUS. However, Brazilian population coverage by OHTs (39.93%) is still much lower than population coverage by physician-nurse centered Family Health Teams (62.62%) [9]. Therefore, considering the expansion of access to services and restructuring of oral health care, oral and oropharyngeal cancer indicators have been positively impacted. However, this association has not been fully evaluated. In view of the foregoing facts, the aim of this study was to analyze the association between late diagnosis of oral and oropharyngeal cancers in Brazil and the contextual indicators of socioeconomic variations and PHC coverage, including individualized assessment of the coverage provided by the OHTs, and assessing the temporal trend of late initial diagnosis.

## Methods

### Study design and population

In this observational study, secondary data were evaluated with a time series analysis model. Data on oral and oropharyngeal cancers cases were obtained from the Hospital Registration Information System (SisRHC, *Sistema de Informações de Cadastro Hospitalar*) of the José Alencar Gomes da Silva National Cancer Institute (INCA), which gathers information from public and private health units and centers that offer cancer treatment in Brazil [10]. All information is in the public domain, however, without patient identification. Therefore, there was no need for project approval by the Ethics Committee on Research with human beings.

INCA considers malignant neoplasms of the lip and oral cavity as being those primarily located in the lips, oral cavity, salivary glands and oropharynx (C00-C10 of the 10th revision of the International Classification of Diseases - ICD-10) [11]. In the literature, there is some variability relative to the inclusion of salivary glands in the category of oral cancer. Many authors defend the exclusion of this structure from the latter category because salivary gland neoplasms show diverse etiological, histological and epidemiological behavior; nevertheless, important sources of global data, such as the Global Burden of Disease Study [12], do include salivary glands in the above-mentioned category. As this work analyzes data from sources in Brazil, the same anatomical sites as those used by INCA, the main authority on the subject, in this country, were included as oral cancers.

The study analysis unit comprised 160 Brazilian municipalities that had at least one case of oral and oropharyngeal cancer, per annum, notified to the RHC-INCA between the years 2000 and 2013.

### Variables and data collection

The outcome variable, risk ratio for late diagnosis, was measured based on the breakdown of items related to the “TNM” system: “tumor size”, “lymph node involvement” and “metastasis”, found in the database as a description of the tumor characteristics at the first hospital visit [10]. “TNM” is an anatomical staging system that describes the anatomical extension of the primary tumor and the involvement of regional lymph nodes and distant metastases [13].

The cases were classified as "0" or "1" in each variable (tumor size, lymph node involvement and metastasis): "0" meant that the tumor staging was less severe: in this case, the diagnosis was attained early; “1” meant that the tumor staging was more severe: in this case, the diagnosis was of the late type. The categories “early stage diagnosis” (0) and “late stage diagnosis” (1) were organized as follows:

- Early stage diagnosis (0): for “size”—tumors classified in the TNM T0, T1 and T2 codes; for “lymph nodes”—tumors classified in N0 code; for “metastases”—tumors classified under M0 code.
- Late diagnosis (1): for “size”—tumors classified in TNM T3 and T4 codes; for “lymph nodes”—tumors classified in N1, N2 and N3 codes; for “metastases”—tumors classified under M1 code. The detailed meaning of these codes is available in the supplementary material (Additional file 1: Table S1).

To generate a record for each municipality, we calculated the risk ratio (RR) for late diagnosis, by dividing the number of late stage diagnoses by the total number of cases with a known diagnosis, per municipality, and for each of the outcome variables separately. The RR varied from "0" to "1"—where "0" would mean that all cases in the city were diagnosed early, and "1" that all cases in the city were diagnosed late.

The mean values of the annual RR of each city (calculated by the arithmetic mean) were the outcome variables in our statistical analysis—the RR considering tumor size, lymph node involvement and metastasis.

The exposure variables for linear regression analysis were:

- Coverage by PHC Oral Health Teams (OHTs)—which indicated the percentage of the municipality covered by these teams.
- Coverage by Family Health Strategy (FHS) teams.
- Coverage by Community Health Agents (CHAs).
- Gini Index, and
- Municipal Human Development Index (M-HDI).

The number of teams in a given year was multiplied by 3,450 (ideal number of people to be cared for by an OHT or FHS, according to the Ministry of Health) [14, 15] and divided by the population of the municipality in the same year, in order to calculate the annual number of persons receiving coverage provided by the OHT and FHS of each municipality. This value was multiplied by 100 to obtain the coverage percentage. The CHA coverage was calculated in practically the same way, considering that the ideal number of people for a CHA would be 575, according to the Ministry of Health [16]. The mean coverage of the municipality represented the arithmetic mean value of the annual coverage between the years 2005 and 2013. The numbers of persons covered by the respective OHTs, FHSs and CHAs were obtained from the website of the Primary Care Department of the Ministry of Health of Brazil.

The numbers of inhabitants of the municipalities in each year were obtained from the Brazilian Institute of Geography and Statistics (IBGE, *Instituto Brasileiro de Geografia e Estatística*) [17].

The Municipal Human Development Index (M-HDI) was obtained from IBGE website [17]. The M-HDI is a vital indicator created by the United Nations to assess the quality of life and economic development of a region, ranging from 0 (without human development) to 1 (full human development).

The Gini index is used to measure social inequality and ranges from 0 (without income inequality) to 1 (total income inequality). For each municipality, this variable was also obtained from the IBGE website. We used data from the year 2010, as they were the only data available for the period of interest for this study [17].

### Statistical analysis

Linear regression models were used to analyze the association between outcomes and exposure variables, for the period of 2005–2013. The cut-off point in the total period of 2000–2013 was necessary for association analyses, as we observed the predominance of notifications with "zeros" referring to the independent variable "OHT coverage" in the years prior to 2005, in all municipalities.

For each result, we created a crude model (result tested in association with each exposure variable separately). Then, according to their *p*-value results, we created the adjusted model (*p* values < 0.25 in the crude model, indicated the variables that were added to the adjusted model). The selection of the best model considered the goodness-of-fit measure of the “R2” model. Results with *p* < 0.05 were considered statistically significant. The normality of the distribution in the result variables was tested by the histogram analysis.

Finally, the temporal trend of RR for late diagnosis was evaluated considering tumor size, lymph node involvement and metastasis in the period of 2000–2013, for all municipalities. In this analysis, a generalized linear regression was performed using the Prais–Winsten method, which allows the correction of the first-order autocorrelation in the analysis of a series of values organized over time. This procedure allowed classifying the rates as ascending (*p* < 0.05 and positive regression coefficient), descending (*p* < 0.05 and negative regression coefficient) or stationary (*p* > 0.05), and quantifying the annual mean values of increase or decrease in the coefficients (annual percentage change—APC). As well as the 95% confidence interval of the coefficients according to the methods used by Antunes and Cardoso [18]. All analyses were carried out using the Stata 14.0 software.

## Results

One hundred and sixty Brazilian municipalities had at least one case per annum of oral and oropharyngeal cancers notified to RHC-INCA between the years 2005 and 2013. Additional information on each of the municipalities is provided in the supplementary material (Additional file 1: Table S2). Table 1 shows the descriptive analysis of the study variables.

**Table 1:** Description of outcome and exposure variables, considering the 160 municipalities analyzed

Variable	Mean (SD^a^)	Median	Minimum	Maximum
*Dependent variables*				
Size (RR^b^)	0.62 (0.12)	0.62	0.25	0.90
Lymph node (RR^b^)	0.55 (0.10)	0.55	0.19	0.81
Metastasis (RR^b^)	0.04 (0.04)	0.03	0.00	0.27
*Independent variables*				
Gini index	0.51 (0.06)	0.50	0.37	0.64
HDI-M^c^	0.76 (0.04)	0.76	0.62	0.86
OHT^d^ coverage (%)	17.34 (20.32)	10.17	0.00	93.77
FHS^e^ coverage (%)	32.94 (24.80)	25.81	0.00	95.71
CHA^f^ coverage (%)	42.65 (31.22)	32.32	0.00	142.21

^a^SD: Standard Deviation

^b^RR: Risk ratio for the most severe diagnosis (RR = 1) at the beginning of the treatment; obtained by dividing the number of cases with late diagnosis by the number of all cases;

^c^M-HDI: Municipal Human Development Index

^d^OHT: Oral Health Team

^e^FHS: Family Health Strategy

^f^CHA: Community Health Agent

The adjusted model showed that, among the contextual variables analyzed, the Gini index was positively associated, while the HDI was negatively associated with the risk ratio for late diagnosis, considering tumor size. That is, the higher the Gini index value (greater social inequality) and the lower the HDI value (less human development), the higher were the number of tumors diagnosed at a late stage, considering tumor size (Table 2).

**Table 2:** Results of the linear regression model for the tumor size outcome of oral and oropharynx cancers

Variables	Crude model					Adjusted model				
	β^a^	95%CI^b^ (−)	95%CI^b^ (+)	*p* value	Adjusted β	β^a^	95%CI^b^ (−)	95%CI^b^ (+)	*p* value	Adjusted β
Gini index	0.24	− 0.07	0.56	0.132	0.11	0.34	0.02	0.66	0.037	0.16
HDI-M^c^	− 0.91	− 1.36	− 0.46	< 0.001	− 0.30	− 0.98	− 1.47	− 0.49	< 0.001	− 0.32
OHT^d^ coverage	< 0.01	< −0.01	< 0.01	0.404	0.06	–	–	–	–	–
FHS^e^ coverage	< 0.01	< 0.01	< 0.01	0.023	0.17	< 0.01	< −0.01	< 0.01	0.372	0.11
CHA^f^ coverage	< 0.01	< 0.01	< 0.01	0.027	0.17	< −0.01	< −0.01	< 0.01	0.673	− 0.05

^a^β: regression coefficient

^b^95% CI: 95% Confidence Interval

^c^ M -HDI: Municipal Human Development Index

^d^OHT: Oral Health Team

^e^FHS: Family Health Strategy

^f^CHA: Community Health Agent

Table 3 shows that among the contextual variables analyzed, the greater the social inequality and the lower the proportion of OHT coverage, the greater was the risk for late diagnosis, as early as in the phase of lymph node involvement.

**Table 3:** Results of the linear regression model for the lymph node outcome of oral and oropharynx cancers

Variables	Crude Model					Adjusted Model				
	β^a^	95%CI^b^ (−)	95%CI^b^ (+)	*p* value	Adjusted β	β^a^	95%CI^b^ (−)	95%CI^b^ (+)	*p* value	Adjusted β
Gini index	0.34	0.06	0.62	0.017	0.18	0.45	0.17	0.74	0.002	0.25
HDI-M^c^	< −0.01	− 0.42	0.40	0.966	< −0.01	–	–	–	–	–
OHT^d^ coverage	< −0.01	< −0.01	< −0.01	0.018	− 0.18	< −0.01	< −0.01	< −0.01	0.002	− 0.24
FHS^e^ coverage	< 0.01	< −0.01	< 0.01	0.867	0.01	–	–	–	–	–
CHA^f^ coverage	< −0.01	< −0.01	< 0.01	0.858	− 0.01	–	–	–	–	–

^a^β: regression coefficient

^b^95% CI: 95% Confidence Interval

^c^ M-HDI: Municipal Human Development Index

^d^OHT: Oral Health Team

^e^FHS: Family Health Strategy

^f^CHA: Community Health Agent

Among the contextual variables analyzed, the greater the social inequality, the higher was the risk of late diagnosis, as early as in the phase of metastasis (Table 4).

**Table 4:** Results of the linear regression model for the metastasis outcome of oral and oropharynx cancers

Variables	Crude model					Adjusted model				
	β^a^	95%CI^b^ (−)	95%CI^b^ (+)	*p* value	adjusted β	β^a^	95%CI^b^ (−)	95%CI^b^ (+)	*p* value	adjusted β
Gini index	0.18	0.06	0.30	0.003	0.23	0.17	0.05	0.29	0.007	0.21
HDI-M	− 0.03	− 0.20	0.14	0.726	− 0.03	–	–	–	–	–
OHT coverage	< −0.01	< −0.01	< 0.01	0.694	− 0.03	–	–	–	–	–
FHS coverage	< 0.01	< −0.01	< 0.01	0.188	0.10	< 0.01	< −0.01	< 0.01	0.453	0.05
CHA coverage	< 0.01	< −0.01	< 0.01	0.473	0.06	–	–	–	–	–

^a^β: regression coefficient

^b^95% CI: 95% Confidence Interval

^c^M-HDI: Municipal Human Development Index

^d^OHT: Oral Health Team

^e^FHS: Family Health Strategy

^f^CHA: Community Health Agent

Table 5 shows the temporal trend of staging of the malignant neoplasms of the oral cavity and oropharynx. During the period evaluated, there was an increase in the number of cases diagnosed at the most advanced stage.

**Table 5:** Trend in risk for advanced stage oral and oropharyngeal cancers: cases reported in the SisRHC-INCA

Outcomes	APC^a^ (%)	95% CI^b^ (−)	95% CI^b^ (+)	*p* value	Trend
Tumor Size	0.46	0.05	0.87	0.030	Ascending
Lymph node	0.99	0.58	1.40	< 0.001	Ascending
Metastasis	4.42	1.14	7.81	0.012	Ascending

^a^APC: Annual percent change

^b^95% CI: 95% Confidence Interval

## Discussion

The findings of this study demonstrated the association between the Gini index and all outcomes, showing the significant contribution of social inequality to the diagnosis of late-stage oral and oropharyngeal cancers. Moreover, there was an association between lower HDI and higher risk ratio for late diagnosis considering tumor size, suggesting the influence of contextual socioeconomic indicators in the early detection of oral and oropharyngeal cancers. The fact that there is an association between socioeconomic indicators and oral and oropharyngeal cancers has been well assessed in the literature [4, 19]. This previously known association, corroborated by this study, requires reflection on pro-equity efforts against this disease [20], and the assessment of existing health policies should also be part of this discussion.

It is noteworthy that coverage by the OHT and the staging of the cancer cases assessed were associated, which indicates that patients in municipalities with greater access to OHT assistance were less likely to receive a diagnosis at advanced stages. The scarcity of epidemiological studies on oral and oropharyngeal cancer staging, in the Brazilian literature, should be noted. Studies analyzing the indicators of these diseases associated with health service availability in the country are even scarcer.

Regarding oral health care in the Unified Health System, PHC is primarily represented by the OHT of the FHS [21]. Our findings indicated the importance of primary care for oral health and its attributes, such as the responsibility for population health, formation of bonds, working with priority groups and in a health care network [22, 23]. These characteristics favor preventive actions over risk factors. Additionally, the expansion of access to dental care for a historically unassisted population, experienced in Brazil since the implementation of the National Oral Health Policy (PNSB) and put into practice by means of the OHT, may have had an impact on the more timely identification of lesions.

Some little explored evidence supported the hypothesis that the restructuring of oral health care in SUS may have had a positive impact on oral cancer. This evidence pointed out access to information about the disease, the demand for dental care and possibility of identifying tumors at earlier stages, especially in risk groups, all of which are improved by the presence of OHTs. Accordingly, people who lived in municipalities with a higher level of PHC coverage were more likely to receive care in advanced health centers [24, 25]. Therefore, it is plausible to establish that the adequate interconnection of health network services (coordinated by PHC), is essential for an effective approach to diagnosing oral or oropharyngeal cancers.

Our results did not indicate that the greater coverage of Family Health Teams, defined as the priority strategy for PHC expansion and qualification, and as the main gateway to SUS [26], contributed to the early diagnosis of oral and oropharyngeal cancers. Therefore, the importance of the need for and expansion of OHT in the country is reinforced. After the publication of the new Primary Care policy in 2017, a movement was observed indicating a reduction in the number of OHTs in Brazil, particularly in the most unequal municipalities in terms of income distribution and larger populations [27]. In this sense, it is worth mentioning the political-economic time that Brazil is going through at present; a time during which the present government, with the justification of the need for austere measures, has promoted the dismantling and precariousness of SUS. This process has also affected oral health care, by deactivation of health units that make up the oral health care network (particularly the Dental Specialty Centers). Considering the austere measures are known to affect SUS as a whole, it is worth taking into account the potential these said measures have to weaken, undermine, and could, in the very short term, lead to a situation of annihilating the greatest social conquest of the Brazilian population – SUS – their life-line to health care.

However, it is essential to consider the natural history of oral or oropharyngeal cancers. Both result from the accumulation of genetic mutations throughout life, which means that it takes many years for them to develop, even in cases of massive exposure to risk factors. Data emerging from evidence have shown that although alcohol and tobacco consumption are the main risk factors for these diseases, the role played by other factors such as socioeconomic disadvantage, genetics, oral health and human papillomavirus (the latter only for oropharyngeal cancer) has become more evident [4, 19]. Therefore, it is understood that the cases analyzed in this study are due to exposure in previous decades.

With regard to the trend analysis, our study showed an upward trend for the risk of being diagnosed at more advanced stages for all outcomes. A few possible interpretive dimensions are available to understand this result. The first corroborated a finding that reflected the improved access to the health care network: the tertiary health centers started to treat patients who would previously not have had access to the public health system, and would have died after going untreated, consequently, without hospital records. During a similar period, it was observed that the mortality rates from oral and oropharyngeal cancers showed a stable or decreasing trend in almost all regions of the country [28], which contributed to this interpretation.

The second point of view concerns the quality of health records in recent years: the correct classification of the most severe cases by primary location is a challenge. Therefore, they could previously have been poorly classified and recorded, due to unclear causes, and were better identified and recorded in the historical series analyzed. This might explain the increase in the number of cases with advanced staging found in this study, mainly when considering that dental surgeons were only included in the FHS teams in 2004 through the implementation of the National Oral Health Policy (PNSB).

There are many obstacles to be faced to achieve consolidation of the Brazilian primary oral health care, mainly with regard to the practical application of its principles and guidelines. While the PNSB advocates a new model of care that emphasizes prevention and health promotion in families and communities, individual and curative treatment remains the hegemonic practice in our health system [20, 23, 29]. Coverage, management, structure and vulnerabilities of the work process have been described in the scientific literature [6, 23, 30]. However, we must emphasize that the increased access to dental care observed in the last decades was unprecedented in the history of the country and that despite some limitations, its pro-equity core seems to prevail [5].

The study limitations include vulnerability of the database due to a low level of completeness of information at the beginning of the series, or the delay in making information available throughout the country at the end of the historical series. Therefore, we chose to perform an ecological study. However, the original internal operational control database validates the data to avoid duplication of records, among others [10]. In this study, the oral cancer classification adopted by INCA was used, i.e., neoplasms classified under the codes C00 to C10 of the International Statistical Classification of Diseases and Problems Related to Health, 10th revision (ICD-10), which is the main cancer surveillance institution in Brazil [11]. However, this classification differs (that is; it includes salivary glands) from the most frequently adopted classifications [31], making it difficult to compare the results of the present study with others that use different standards to classify these neoplasms. It is important to note that the cases analyzed did not account for all cases of oral and oropharyngeal cancers treated in the hospital environment in Brazil, as the database used included only the records sent to SisRHC-INCA. Based on the estimates of new cancer cases in Brazil, which are published by INCA every six months, we verified that the SisRHC-INCA covers about 66% of the estimated cases, considering the years 2010 to 2018 (unpublished data). Additionally, the coverage was calculated considering the number of people living and registered in the region, and not the people using the services. Nevertheless, it is the best and most complete national database available on the pathologies evaluated since cancer notification is not mandatory in Brazil. The other limitations are intrinsic to the methodological design of the study, which have been widely discussed in the literature [28], such as the impediments in the processing of aggregated data and the lack of individual data, which are necessary for a more detailed investigation.

## Conclusions

We concluded that, during the evaluated period, there was an increase in the number of cases diagnosed at the most advanced stage. Furthermore, we observed that the higher level of coverage of OHT services was associated with the diagnosis of oral and oropharyngeal cancers in the early stages. This suggested a potential increase in the chances of treatment and cure. However, although the PNSB has been implemented for over 15 years, there is less than 50% OHT coverage in the Brazilian territory. It is necessary to strengthen the growing qualification of this strategy and evaluate its effects, with the aim of enabling well-directed and effective efforts against oral and oropharyngeal cancers.

The association between higher levels of social inequalities and an increase in the proportion of late diagnosis of oral and oropharyngeal cancers observed in this study reinforces the need to discuss the reduction of inequalities in different social groups. This process requires the creation and strengthening of public health policies with the aim of not only the prevention of individual behaviors related to oral or oropharyngeal cancers, but also of addressing the socioeconomic and cultural determinants that give rise to the origin of these behaviors. Only through adequate planning and investment will it be possible to reverse the upward trend in the number of cases diagnosed at advanced stages, found in this study.

## Supplementary Information

**Additional file 1**. Table S1 Meaning of TNM codes and their distribution according to the staging categories used in the study. Table S2 Sample of 160 municipalities, showing medium risk relative to the more severe outcome in relation to the less severe one ("T" represents tumor size, "N" represents lymph node involvement and "M" represents the presence of distant metastasis).

## Data Availability

All data generated or analyzed during this study are included in this published article [and its supplementary information files].

## Abbreviations

CHA: Community Health Agents
FHS: Family Health Strategy
IBGE: Brazilian Institute of Geography and Statistics
INCA: National Cancer Institute José Alencar Gomes da Silva
M-HDI: Municipal Human Development Index
OHT: Oral Health Team
PHC: Primary Health Care
PNSB: National Oral Health Policy
RR: Risk ratio
SD: Standard Deviation
SUS: Unified Health System
