# Supplementary material for: Inclusion of oral health teams in primary health care promotes early diagnosis of oral and oropharyngeal cancers: a nationwide study
Source: BMC Oral Health. 2021 Jun 18;21:312. doi: 10.1186/s12903-021-01664-3 (PMC8212463; doi:10.1186/s12903-021-01664-3)
Supplement: Supplementary file 1 — Additional file 1. Table S1 Meaning of TNM codes and their distribution according to the staging categories used in the study. Table S2 Sample of 160 municipalities, showing medium risk relative to the more severe outcome in relation to the less severe one ("T" represents tumor size, "N" represents lymph node involvement and "M" represents the presence of distant metastasis). [file 12903_2021_1664_MOESM1_ESM.docx]

**Inclusion of Oral Health Teams in Primary Health Care promotes the early diagnosis of oral and oropharyngeal cancers: a nationwide study**

Deborah Gomes de Miranda Vargas, Livia Fernandes Probst, Amanda Ramos da Cunha, Elaine Pereira da Silva Tagliaferro, Edílson José Zafalon, Paulo Zárate Pereira, Alessandro Diogo De Carli

**Supplementary Material**

**Table S1** Meaning of TNM codes and their distribution according to the staging categories used in the study

| **Tumor variables** | **Early stage diagnosis (0)** | **Late stage diagnosis (1)** |
| --- | --- | --- |
| Size | T0: No evidence of primary tumor.  T1: Tumor with 2cm or less in largest dimension.  T2: Tumor larger than 2cm and up to 4cm in its largest dimension. | T3: Tumor larger than 4cm in its largest dimension.  T4: Tumor invading adjacent structures. |
| Lymph node | N0: Absence of regional lymph node metastasis. | N1: Metastasis in a single homolateral lymph node, with 3cm or less in largest dimension.  N2 (oral cavity): Metastasis in a single homolateral lymph node, with more than 3cm and up to 6cm in its largest dimension; or in bilateral or contralateral lymph nodes, none larger than 6cm in their largest dimension.  N2 (oropharynx): All N2 criteria for oral cavity plus metastasis in multiple homolateral lymph nodes, none larger than 6cm.  N3: Lymph node metastasis larger than 6cm in its largest dimension. |
| Metastasis | M0: Absence of distant metastasis. | M1: Distant metastasis. |

**Table S2** Sample of 160 municipalities, showing medium risk relative to the more severe outcome in relation to the less severe one

| **City*** | **T**  **Tumor size** | **N**  **Lymph node involvement** | **M**  **Distant metastasis** | **OHT**  **(%)** | **FHS**  **(%)** | **CHA**  **(%)** | **HDI-M** | **Gini Index** |
| --- | --- | --- | --- | --- | --- | --- | --- | --- |
| City_001 | 0.56 | 0.53 | 0.00 | 48.97 | 12.44 | 41.24 | 0.79 | 0.52 |
| City_002 | 0.68 | 0.61 | 0.04 | 0.90 | 12.79 | 13.69 | 0.81 | 0.47 |
| City_003 | 0.76 | 0.62 | 0.05 | 23.82 | 43.89 | 45.11 | 0.76 | 0.49 |
| City_004 | 0.64 | 0.62 | 0.06 | 32.43 | 63.57 | 58.55 | 0.79 | 0.54 |
| City_005 | 0.66 | 0.42 | 0.11 | 67.44 | 77.38 | 136.59 | 0.75 | 0.57 |
| City_006 | 0.90 | 0.43 | 0.11 | 52.68 | 71.40 | 106.23 | 0.65 | 0.56 |
| City_007 | 0.44 | 0.51 | 0.03 | 14.35 | 26.12 | 30.31 | 0.81 | 0.50 |
| City_008 | 0.51 | 0.55 | 0.03 | 31.54 | 48.98 | 43.68 | 0.78 | 0.48 |
| City_009 | 0.59 | 0.50 | 0.02 | 21.67 | 39.35 | 27.94 | 0.81 | 0.50 |
| City_010 | 0.39 | 0.36 | 0.00 | 16.33 | 17.76 | 19.73 | 0.77 | 0.48 |
| City_011 | 0.59 | 0.56 | 0.06 | 41.82 | 60.93 | 61.14 | 0.74 | 0.57 |
| City_012 | 0.64 | 0.70 | 0.14 | 22.51 | 68.09 | 79.74 | 0.77 | 0.60 |
| City_013 | 0.47 | 0.37 | 0.00 | 26.16 | 35.13 | 34.32 | 0.75 | 0.43 |
| City_014 | 0.47 | 0.40 | 0.02 | 0.00 | 0.00 | 12.55 | 0.79 | 0.45 |
| City_015 | 0.59 | 0.43 | 0.03 | 8.63 | 19.88 | 21.64 | 0.79 | 0.49 |
| City_016 | 0.60 | 0.59 | 0.02 | 0.43 | 1.47 | 1.45 | 0.79 | 0.62 |
| City_017 | 0.50 | 0.31 | 0.07 | 24.61 | 24.61 | 69.60 | 0.76 | 0.50 |
| City_018 | 0.53 | 0.53 | 0.05 | 4.70 | 6.82 | 8.80 | 0.80 | 0.56 |
| City_019 | 0.55 | 0.50 | 0.02 | 42.61 | 47.58 | 52.21 | 0.78 | 0.51 |
| City_020 | 0.81 | 0.56 | 0.04 | 05.08 | 18.96 | 36.06 | 0.68 | 0.46 |
| City_021 | 0.70 | 0.61 | 0.04 | 30.84 | 73.07 | 53.35 | 0.81 | 0.61 |
| City_022 | 0.77 | 0.68 | 0.07 | 10.69 | 37.37 | 79.02 | 0.75 | 0.48 |
| City_023 | 0.63 | 0.47 | 0.00 | 0.00 | 28.29 | 48.57 | 0.78 | 0.43 |
| City_024 | 0.52 | 0.48 | 0.04 | 0.00 | 0.00 | 0.00 | 0.78 | 0.49 |
| City_025 | 0.45 | 0.57 | 0.03 | 20.28 | 28.36 | 29.90 | 0.80 | 0.56 |
| City_026 | 0.33 | 0.44 | 0.00 | 0.00 | 15.77 | 10.51 | 0.74 | 0.42 |
| City_027 | 0.71 | 0.41 | 0.00 | 91.64 | 91.64 | 137.17 | 0.68 | 0.56 |
| City_028 | 0.25 | 0.45 | 0.01 | 29.58 | 74.33 | 82.01 | 0.72 | 0.59 |
| City_029 | 0.67 | 0.67 | 0.02 | 17.08 | 36.07 | 22.49 | 0.81 | 0.58 |
| City_030 | 0.67 | 0.57 | 0.19 | 28.35 | 28.6 | 96.83 | 0.78 | 0.57 |
| City_031 | 0.44 | 0.19 | 0.00 | 20.18 | 20.18 | 17.95 | 0.75 | 0.42 |
| City_032 | 0.71 | 0.71 | 0.05 | 0.00 | 0.00 | 0.28 | 0.75 | 0.50 |
| City_033 | 0.77 | 0.66 | 0.05 | 01.07 | 10.79 | 31.73 | 0.72 | 0.47 |
| City_034 | 0.50 | 0.42 | 0.00 | 24.58 | 24.58 | 33.01 | 0.73 | 0.51 |
| City_035 | 0.43 | 0.57 | 0.03 | 53.2 | 56.24 | 50.59 | 0.78 | 0.47 |
| City_036 | 0.63 | 0.50 | 0.00 | 0.00 | 0.00 | 86.07 | 0.76 | 0.46 |
| City_037 | 0.68 | 0.61 | 0.04 | 3.46 | 49.07 | 39.22 | 0.76 | 0.50 |
| City_038 | 0.60 | 0.55 | 0.03 | 3.61 | 13.89 | 13.17 | 0.78 | 0.59 |
| City_039 | 0.51 | 0.62 | 0.04 | 6.25 | 52.84 | 69.18 | 0.79 | 0.49 |
| City_040 | 0.74 | 0.50 | 0.00 | 20.03 | 20.63 | 18.8 | 0.79 | 0.54 |
| City_041 | 0.79 | 0.61 | 0.03 | 17.99 | 41.79 | 44.13 | 0.74 | 0.47 |
| City_042 | 0.58 | 0.53 | 0.03 | 37.03 | 56.84 | 61.15 | 0.76 | 0.46 |
| City_043 | 0.61 | 0.65 | 0.02 | 22.17 | 23.5 | 19.85 | 0.76 | 0.48 |
| City_044 | 0.61 | 0.54 | 0.08 | 60.11 | 64.78 | 78.39 | 0.75 | 0.53 |
| City_045 | 0.59 | 0.47 | 0.00 | 0.00 | 2.94 | 21.3 | 0.78 | 0.45 |
| City_046 | 0.80 | 0.65 | 0.02 | 14.04 | 21.15 | 29.5 | 0.71 | 0.49 |
| City_047 | 0.66 | 0.57 | 0.03 | 11.74 | 18.26 | 23.35 | 0.74 | 0.49 |
| City_048 | 0.72 | 0.62 | 0.02 | 18.19 | 49.92 | 95.63 | 0.71 | 0.61 |
| City_049 | 0.65 | 0.51 | 0.01 | 43.83 | 56.28 | 66.38 | 0.80 | 0.49 |
| City_050 | 0.70 | 0.57 | 0.04 | 3.41 | 10.49 | 9.64 | 0.74 | 0.46 |
| City_051 | 0.57 | 0.56 | 0.11 | 28.45 | 77.86 | 73.12 | 0.85 | 0.55 |
| City_052 | 0.71 | 0.64 | 0.06 | 24.81 | 31.82 | 39.73 | 0.75 | 0.63 |
| City_053 | 0.56 | 0.41 | 0.09 | 0.00 | 5.34 | 4.46 | 0.78 | 0.47 |
| City_054 | 0.64 | 0.49 | 0.01 | 17.16 | 20.97 | 23.30 | 0.80 | 0.55 |
| City_055 | 0.64 | 0.57 | 0.01 | 6.81 | 15.63 | 19.55 | 0.75 | 0.52 |
| City_056 | 0.69 | 0.60 | 0.03 | 09.06 | 20.89 | 30.48 | 0.76 | 0.53 |
| City_057 | 0.79 | 0.70 | 0.02 | 1.74 | 15.71 | 11.12 | 0.76 | 0.43 |
| City_058 | 0.75 | 0.74 | 0.00 | 2.15 | 76.59 | 87.60 | 0.70 | 0.45 |
| City_059 | 0.39 | 0.47 | 0.00 | 0.00 | 10.93 | 10.93 | 0.75 | 0.45 |
| City_060 | 0.53 | 0.47 | 0.00 | 0.00 | 0.00 | 14.19 | 0.73 | 0.37 |
| City_061 | 0.62 | 0.46 | 0.03 | 7.49 | 15.36 | 11.02 | 0.79 | 0.49 |
| City_062 | 0.73 | 0.65 | 0.08 | 1.92 | 3.19 | 19.43 | 0.74 | 0.48 |
| City_063 | 0.43 | 0.53 | 0.07 | 19.48 | 24.93 | 32.2 | 0.76 | 0.50 |
| City_064 | 0.70 | 0.63 | 0.00 | 33.04 | 52.84 | 73.99 | 0.73 | 0.54 |
| City_065 | 0.70 | 0.70 | 0.04 | 6.78 | 07.06 | 21.79 | 0.74 | 0.47 |
| City_066 | 0.42 | 0.42 | 0.02 | 1.55 | 7.63 | 7.91 | 0.74 | 0.45 |
| City_067 | 0.77 | 0.64 | 0.00 | 1.64 | 5.52 | 5.67 | 0.71 | 0.46 |
| City_068 | 0.52 | 0.52 | 0.00 | 0.00 | 0.00 | 0.99 | 0.77 | 0.54 |
| City_069 | 0.55 | 0.52 | 0.00 | 47.25 | 61.36 | 54.49 | 0.77 | 0.53 |
| City_070 | 0.53 | 0.53 | 0.02 | 9.86 | 17.62 | 17.43 | 0.78 | 0.53 |
| City_071 | 0.73 | 0.50 | 0.00 | 48.32 | 56.88 | 121.26 | 0.65 | 0.55 |
| City_072 | 0.47 | 0.39 | 0.02 | 46.84 | 59.60 | 74.46 | 0.78 | 0.46 |
| City_073 | 0.61 | 0.48 | 0.13 | 0.00 | 41.26 | 48.05 | 0.74 | 0.51 |
| City_074 | 0.53 | 0.38 | 0.01 | 0.00 | 30.66 | 45.10 | 0.78 | 0.46 |
| City_075 | 0.50 | 0.50 | 0.00 | 89.09 | 86.46 | 98.39 | 0.76 | 0.63 |
| City_076 | 0.47 | 0.30 | 0.00 | 14.25 | 15.70 | 22.90 | 0.78 | 0.46 |
| City_077 | 0.77 | 0.39 | 0.06 | 72.19 | 84.77 | 116.00 | 0.68 | 0.57 |
| City_078 | 0.72 | 0.70 | 0.06 | 6.84 | 8.30 | 27.05 | 0.82 | 0.54 |
| City_079 | 0.64 | 0.54 | 0.05 | 19.01 | 28.80 | 36.44 | 0.74 | 0.48 |
| City_080 | 0.60 | 0.49 | 0.00 | 0.00 | 34.94 | 85.20 | 0.76 | 0.44 |
| City_081 | 0.76 | 0.70 | 0.27 | 12.69 | 27.67 | 23.64 | 0.72 | 0.64 |
| City_082 | 0.63 | 0.63 | 0.04 | 8.81 | 26.68 | 32.60 | 0.79 | 0.58 |
| City_083 | 0.75 | 0.75 | 0.07 | 10.23 | 32.52 | 45.02 | 0.74 | 0.63 |
| City_084 | 0.74 | 0.53 | 0.09 | 58.96 | 85.14 | 65.22 | 0.69 | 0.44 |
| City_085 | 0.60 | 0.67 | 0.00 | 29.46 | 51.42 | 104.62 | 0.62 | 0.57 |
| City_086 | 0.51 | 0.46 | 0.04 | 48.90 | 48.9 | 76.61 | 0.80 | 0.51 |
| City_087 | 0.57 | 0.48 | 0.03 | 1.13 | 20.04 | 26.06 | 0.77 | 0.53 |
| City_088 | 0.55 | 0.62 | 0.02 | 13.23 | 26.72 | 29.77 | 0.77 | 0.46 |
| City_089 | 0.52 | 0.57 | 0.00 | 12.75 | 25.5 | 109.33 | 0.75 | 0.47 |
| City_090 | 0.65 | 0.41 | 0.00 | 9.38 | 16.85 | 20.71 | 0.76 | 0.47 |
| City_091 | 0.60 | 0.48 | 0.08 | 16.84 | 23.51 | 27.92 | 0.76 | 0.54 |
| City_092 | 0.48 | 0.39 | 0.02 | 17.26 | 21.27 | 21.43 | 0.77 | 0.42 |
| City_093 | 0.68 | 0.55 | 0.07 | 36.53 | 54.71 | 62.19 | 0.77 | 0.54 |
| City_094 | 0.69 | 0.56 | 0.00 | 83.18 | 87.08 | 75.88 | 0.73 | 0.52 |
| City_095 | 0.79 | 0.62 | 0.02 | 4.11 | 21.91 | 25.28 | 0.71 | 0.51 |
| City_096 | 0.59 | 0.47 | 0.03 | 0.00 | 2.82 | 3.87 | 0.75 | 0.45 |
| City_097 | 0.48 | 0.42 | 0.00 | 7.19 | 13.04 | 14.56 | 0.77 | 0.44 |
| City_098 | 0.68 | 0.65 | 0.06 | 0.00 | 0.70 | 16.41 | 0.78 | 0.55 |
| City_099 | 0.59 | 0.51 | 0.00 | 04.09 | 13.31 | 33.55 | 0.78 | 0.51 |
| City_100 | 0.78 | 0.52 | 0.04 | 22.95 | 65.72 | 90.69 | 0.74 | 0.51 |
| City_101 | 0.66 | 0.68 | 0.12 | 34.23 | 58.76 | 68.44 | 0.76 | 0.40 |
| City_102 | 0.42 | 0.38 | 0.00 | 25.47 | 63.63 | 61.79 | 0.75 | 0.46 |
| City_103 | 0.61 | 0.55 | 0.07 | 29.77 | 55.68 | 60.68 | 0.73 | 0.43 |
| City_104 | 0.66 | 0.57 | 0.04 | 0.00 | 27.64 | 28.2 | 0.78 | 0.53 |
| City_105 | 0.54 | 0.54 | 0.00 | 6.30 | 6.30 | 6.40 | 0.74 | 0.50 |
| City_106 | 0.67 | 0.61 | 0.26 | 1.74 | 29.25 | 22.41 | 0.74 | 0.56 |
| City_107 | 0.69 | 0.64 | 0.00 | 17.1 | 74.37 | 69.8 | 0.76 | 0.47 |
| City_108 | 0.73 | 0.63 | 0.02 | 39.52 | 45.71 | 47.3 | 0.77 | 0.52 |
| City_109 | 0.45 | 0.39 | 0.02 | 6.14 | 27.08 | 26.4 | 0.78 | 0.54 |
| City_110 | 0.64 | 0.59 | 0.07 | 0.25 | 73.41 | 67.28 | 0.80 | 0.52 |
| City_111 | 0.44 | 0.59 | 0.04 | 0.00 | 3.97 | 81.01 | 0.72 | 0.42 |
| City_112 | 0.68 | 0.64 | 0.00 | 5.88 | 21.46 | 19.59 | 0.77 | 0.45 |
| City_113 | 0.64 | 0.39 | 0.00 | 10.12 | 10.12 | 12.86 | 0.75 | 0.46 |
| City_114 | 0.72 | 0.66 | 0.03 | 14.72 | 59.98 | 71.43 | 0.75 | 0.51 |
| City_115 | 0.58 | 0.55 | 0.03 | 0.00 | 68.45 | 76.12 | 0.75 | 0.49 |
| City_116 | 0.60 | 0.54 | 0.00 | 15.34 | 20.88 | 21.74 | 0.81 | 0.54 |
| City_117 | 0.65 | 0.65 | 0.04 | 9.76 | 9.76 | 71.94 | 0.74 | 0.44 |
| City_118 | 0.82 | 0.62 | 0.03 | 3.21 | 40.11 | 50.20 | 0.68 | 0.41 |
| City_119 | 0.47 | 0.48 | 0.09 | 4.29 | 14.00 | 32.45 | 0.80 | 0.55 |
| City_120 | 0.57 | 0.51 | 0.03 | 14.34 | 18.51 | 21.22 | 0.80 | 0.51 |
| City_121 | 0.72 | 0.58 | 0.01 | 6.19 | 15.76 | 21.86 | 0.80 | 0.64 |
| City_122 | 0.70 | 0.48 | 0.04 | 5.89 | 67.11 | 84.48 | 0.80 | 0.46 |
| City_123 | 0.28 | 0.81 | 0.00 | 5.55 | 41.43 | 43.48 | 0.74 | 0.52 |
| City_124 | 0.67 | 0.70 | 0.04 | 1.76 | 16.04 | 18.89 | 0.73 | 0.47 |
| City_125 | 0.67 | 0.57 | 0.02 | 10.8 | 16.47 | 31.09 | 0.76 | 0.64 |
| City_126 | 0.62 | 0.72 | 0.02 | 0.00 | 0.00 | 3.37 | 0.78 | 0.42 |
| City_127 | 0.58 | 0.54 | 0.00 | 24.65 | 24.65 | 51.57 | 0.76 | 0.46 |
| City_128 | 0.8 | 0.57 | 0.08 | 0.00 | 62.98 | 56.84 | 0.72 | 0.45 |
| City_129 | 0.65 | 0.44 | 0.00 | 93.77 | 95.71 | 108.61 | 0.63 | 0.48 |
| City_130 | 0.61 | 0.60 | 0.06 | 3.71 | 13.98 | 18.54 | 0.81 | 0.54 |
| City_131 | 0.56 | 0.63 | 0.05 | 1.66 | 9.58 | 30.01 | 0.84 | 0.56 |
| City_132 | 0.46 | 0.55 | 0.02 | 06.08 | 18.85 | 42.27 | 0.81 | 0.55 |
| City_133 | 0.45 | 0.56 | 0.10 | 16.91 | 46.48 | 38.84 | 0.86 | 0.55 |
| City_134 | 0.56 | 0.59 | 0.03 | 14.21 | 21.05 | 20.26 | 0.81 | 0.50 |
| City_135 | 0.62 | 0.55 | 0.00 | 9.55 | 32.03 | 38.06 | 0.8 | 0.52 |
| City_136 | 0.77 | 0.57 | 0.00 | 7.95 | 32.8 | 32.69 | 0.76 | 0.42 |
| City_137 | 0.65 | 0.62 | 0.07 | 0.00 | 60.53 | 57.34 | 0.81 | 0.45 |
| City_138 | 0.53 | 0.40 | 0.02 | 8.19 | 16.27 | 18.59 | 0.80 | 0.51 |
| City_139 | 0.71 | 0.66 | 0.00 | 0.00 | 0.79 | 9.99 | 0.81 | 0.56 |
| City_140 | 0.58 | 0.48 | 0.02 | 8.94 | 28.42 | 21.60 | 0.74 | 0.46 |
| City_141 | 0.62 | 0.57 | 0.04 | 4.23 | 26.78 | 29.42 | 0.80 | 0.65 |
| City_142 | 0.76 | 0.71 | 0.12 | 4.33 | 15.52 | 14.62 | 0.77 | 0.51 |
| City_143 | 0.67 | 0.66 | 0.04 | 0.00 | 8.13 | 14.88 | 0.77 | 0.49 |
| City_144 | 0.74 | 0.68 | 0.10 | 10.64 | 22.25 | 30.96 | 0.74 | 0.49 |
| City_145 | 0.45 | 0.36 | 0.04 | 0.00 | 1.66 | 12.88 | 0.76 | 0.47 |
| City_146 | 0.80 | 0.59 | 0.02 | 17.03 | 43.55 | 48.71 | 0.76 | 0.54 |
| City_147 | 0.56 | 0.56 | 0.06 | 6.61 | 29.07 | 29.96 | 0.73 | 0.45 |
| City_148 | 0.57 | 0.56 | 0.01 | 1.16 | 8.74 | 08.03 | 0.80 | 0.53 |
| City_149 | 0.72 | 0.64 | 0.01 | 15.28 | 18.14 | 28.36 | 0.76 | 0.47 |
| City_150 | 0.66 | 0.61 | 0.05 | 7.92 | 13.34 | 19.86 | 0.77 | 0.49 |
| City_151 | 0.56 | 0.54 | 0.06 | 7.55 | 17.49 | 22.04 | 0.77 | 0.49 |
| City_152 | 0.56 | 0.42 | 0.04 | 0.00 | 0.62 | 0.62 | 0.75 | 0.47 |
| City_153 | 0.60 | 0.53 | 0.04 | 10.28 | 15.34 | 11.03 | 0.80 | 0.52 |
| City_154 | 0.71 | 0.48 | 0.01 | 72.59 | 94.64 | 100.82 | 0.75 | 0.62 |
| City_155 | 0.73 | 0.63 | 0.00 | 71.31 | 87.16 | 142.21 | 0.80 | 0.48 |
| City_156 | 0.78 | 0.73 | 0.03 | 17.59 | 60.61 | 57.53 | 0.72 | 0.50 |
| City_157 | 0.83 | 0.55 | 0.18 | 4.52 | 35.97 | 34.22 | 0.69 | 0.44 |
| City_158 | 0.75 | 0.62 | 0.05 | 16.38 | 24.58 | 26.61 | 0.80 | 0.57 |
| City_159 | 0.69 | 0.62 | 0.09 | 34.31 | 72.61 | 62.82 | 0.84 | 0.61 |
| City_160 | 0.54 | 0.46 | 0.00 | 19.07 | 35.13 | 53.78 | 0.79 | 0.50 |

Data from 2005 to 2013

OHT: Oral Health Team

FHS: Family Health Strategy

CHA: Community Health Agents

M-HDI: Municipal Human Development Index

*In order to avoid exposure of the municipalities analyzed, we coded their names.
